# Supplementary material for: The role of endogenous and exogenous mechanisms in the formation of R&D networks
Source: Sci Rep. 2014 Jul 14;4:5679. doi: 10.1038/srep05679 (PMC4097357; doi:10.1038/srep05679)
Supplement: Supplementary Information [file srep05679-s1.pdf]

Supplementary Information
to
The role of endogenous and exogenous mechanisms in the formation of R&D networks

Mario V. Tomasello *[1], Nicola Perra[2], Claudio J. Tessone[1], Márton Karsai[3], and Frank Schweitzer[1]

[1]Chair of Systems Design, Department of Management, Technology and Economics (D-MTEC), ETH Zurich, Weinbergstrasse 56/58, 8092 Zurich, Switzerland
[2]Laboratory for the Modeling of Biological and Socio-technical Systems, Northeastern University, Boston, MA 02115, USA
[3]Laboratoire de l'Informatique du Parallélisme, INRIA-UMR 5668, IXXI, ENS de Lyon, 69364 Lyon, France

Dataset details

The typical dispersed and right-skewed distribution of partners per alliance is a universal feature across industrial sectors. In Fig. 1 we show such distribution for the nine largest sectors reported in the dataset.

Empirical firm activities are robust with respect to the time t at which they are measured. In Fig. 2, we show that shifting the time window – of any length Δt – along the 26 year observation period of the dataset does not affect the results.

In addition, we find that the activity distribution is robust to the sectoral classification of the firms. In Fig. 3 we show the empirical firm activity distributions (computed on four different time windows) for the nine largest sectors reported in the dataset.

Numerical simulations results

For each of the 684,000 computer simulations we run, we test the resulting generated R&D network with respect to three properties: average degree $\langle k \rangle$, average path length $\langle l \rangle$ and global clustering coefficient C. In Fig. 4 we show how these three quantities are distributed across all the 684,000 realizations and we compare them with the observed values $\langle k \rangle^{OBS}$, $\langle l \rangle^{OBS}$ and C^{OBS}.

We find that the global clustering coefficient and the average path length distributions are peaked around the observed values. However, the average degree distribution does not display any peak, despite being relatively narrow and centered around the real value (note the values on the x-axis in Fig. 4). The fact that these three distributions are centered around the real values testifies that our model well captures the topology of the observed network for a large set of free parameters, despite we have imposed only a few features of the network (number of nodes N and alliances E, and the distributions of node activities a_i and partners per alliance m). At the same time, the distributions of $\langle k \rangle$, $\langle l \rangle$ and C are not excessively narrow, showing that the we can meaningfully perform an exploration – and consequently a fit – of the free parameters of our model.

*mtomasello@ethz.ch

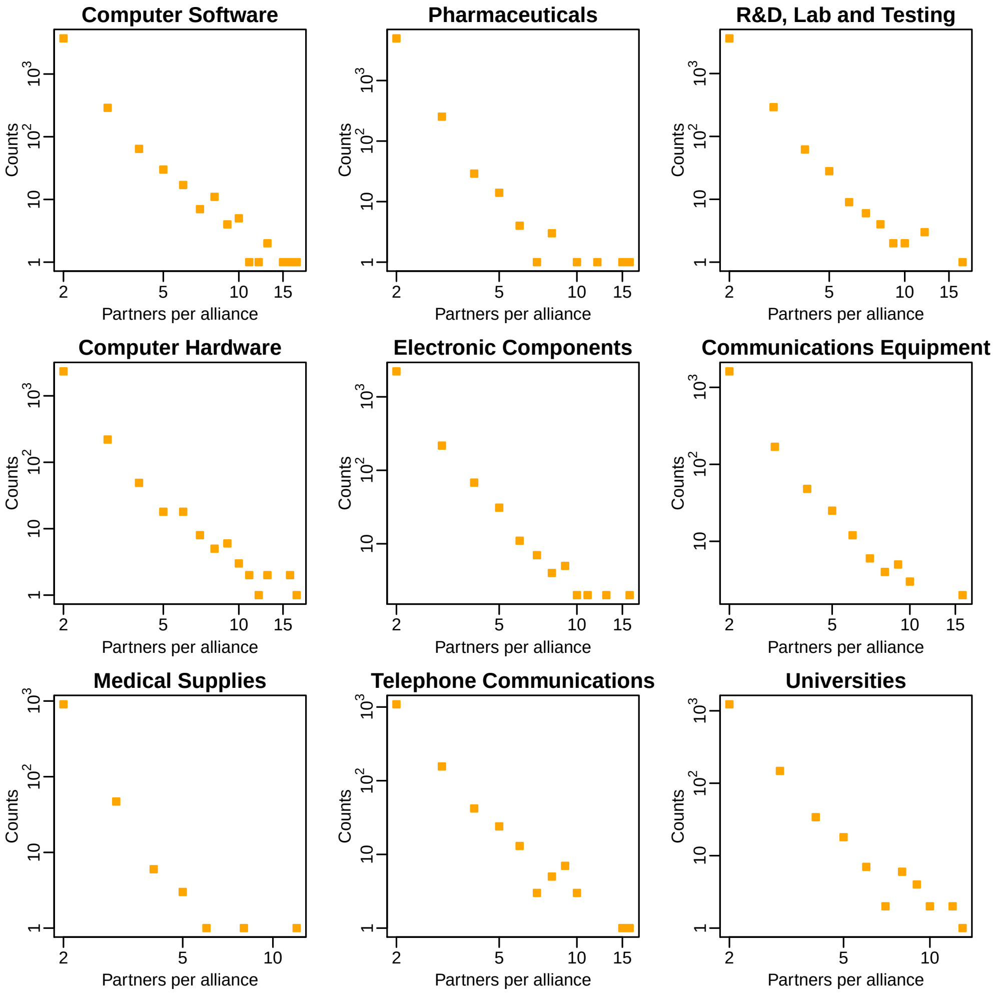

Figure 1: Distribution of the number of partners per alliance for the nine largest industrial sectors, as measured from the SDC dataset.

The error threshold value ϵ^0 we impose for the computation of the Likelihood score influences the number of points in the parameter space that fulfill our matching criteria. Obviously, by decreasing ϵ^0, we observe a smaller number of points displaying high likelihood scores, as we could expect, because a better representation of reality is required. In Fig. 5 we show the Likelihood scores of every point in the parameter space for six different values of ϵ^0, ranging from 1% to 10%. For our analysis, we take a conservative approach and fix $\epsilon^0 = 2\%$.

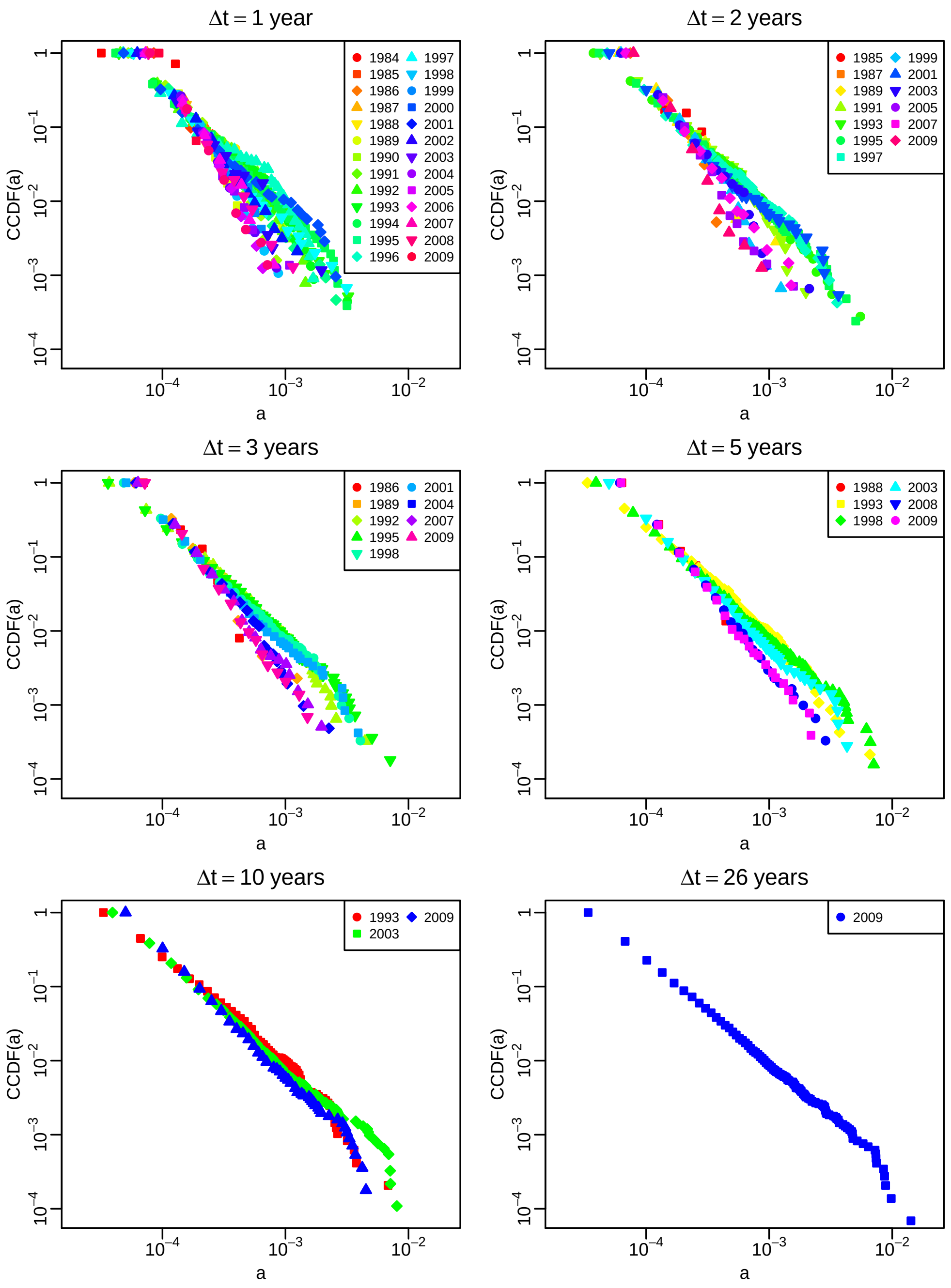

Figure 2: Complementary cumulative distribution function (CCDF) of the empirical firm activities, measured on the SDC dataset with 6 different time windows Δt of 1, 2, 3, 5, 10 and 26 years. When the time window is shorter than 26 years, we shift such time window along the observation period and show the corresponding activity CCDF.

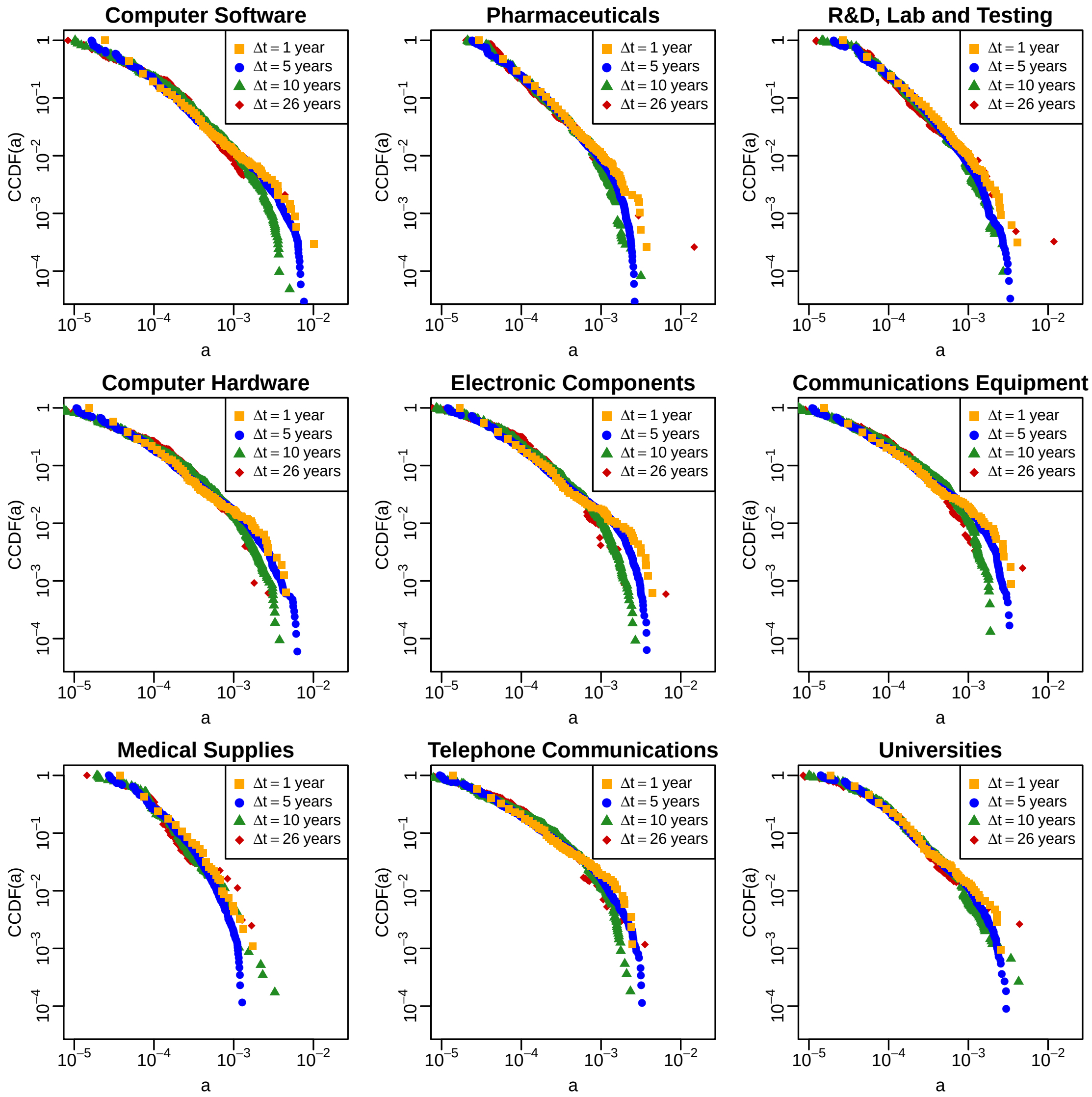

Figure 3: Complementary cumulative distribution function (CCDF) of the empirical firm activities, measured for the nine largest industrial sectors in the SDC dataset.

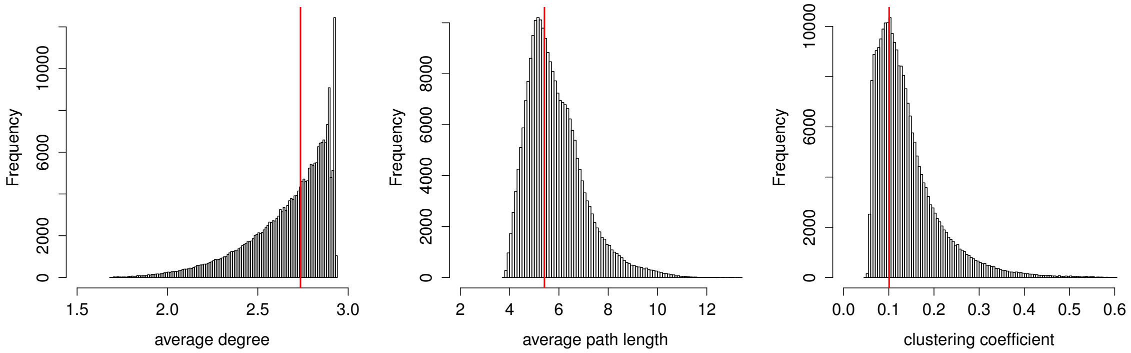

Figure 4: Distributions of average degree $\langle k \rangle$, average path length $\langle l \rangle$ and global clustering coefficient C across all 684,000 runs of our model (each of the 3,420 points in the parameter space has been explored 200 times). The vertical red lines represent the observed values $\langle k \rangle^{OBS}$, $\langle l \rangle^{OBS}$ and C^{OBS} in the empirical R&D network.

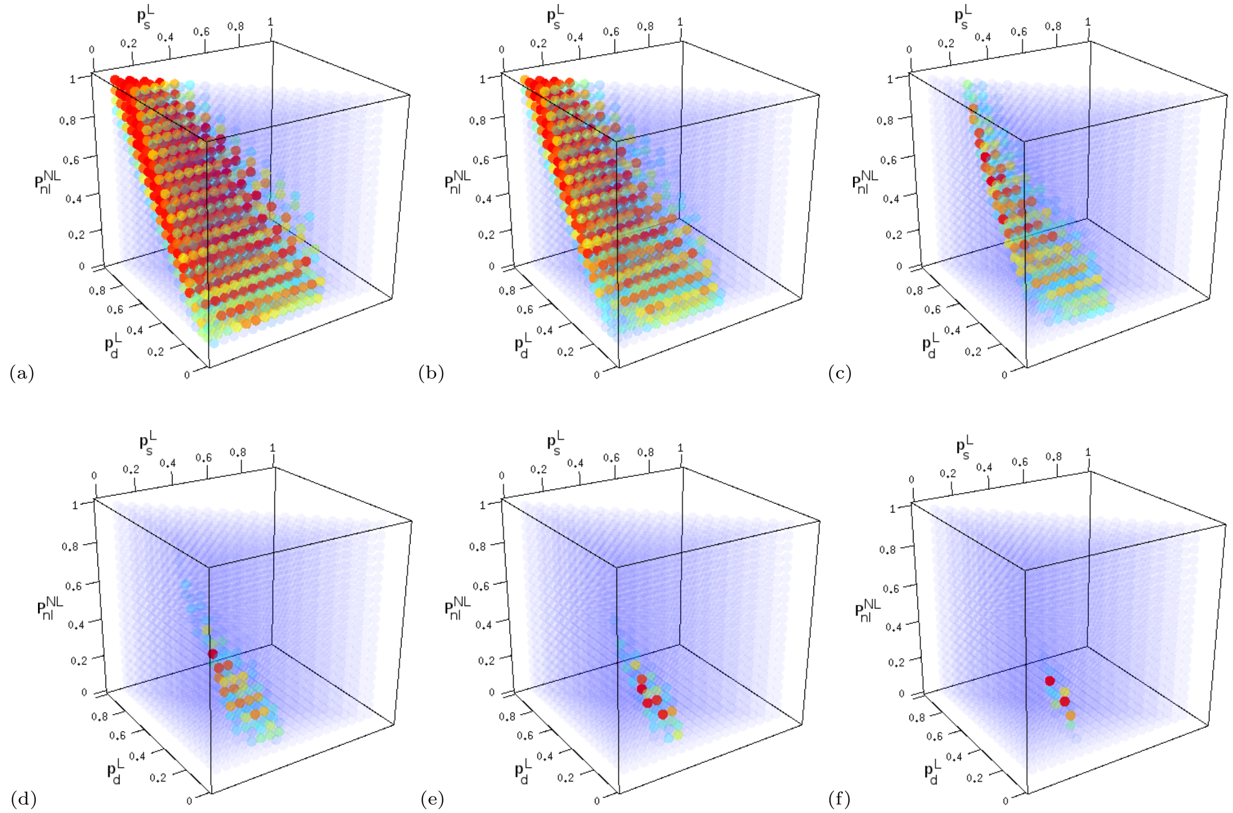

Figure 5: Likelihood scores for all points in the parameter space, for ϵ^0 equal to 10% (a), 8% (b), 5% (c), 3% (d), 2% (e) and 1% (f).
